# iMOVE: Intensive Mobility training with Variability and Error compared to conventional rehabilitation for young children with cerebral palsy: the protocol for a single blind randomized controlled trial

**DOI:** 10.1186/s12887-018-1303-8

**Published:** 2018-10-16

**Authors:** Laura A. Prosser, Samuel R. Pierce, Timothy R. Dillingham, Judy C. Bernbaum, Abbas F. Jawad

**Affiliations:** 1 Division of Rehabilitation Medicine, The Children’s Hospital of Philadelphia, 3401 Civic Center Blvd, Philadelphia, PA 19104 USA; 2 Department of Pediatrics, Perelman School of Medicine, University of Pennsylvania, 3401 Civic Center Blvd, Philadelphia, PA 19104 USA; 3 Widener University, Institute for Physical Therapy Education, One University Place, Chester, PA 19013 USA; 4 Department of Physical Medicine and Rehabilitation, Perelman School of Medicine, University of Pennsylvania, Philadelphia, PA 19104 USA; 5 Division of General Pediatrics, The Children’s Hospital of Philadelphia, Philadelphia, PA 19104 USA

**Keywords:** Cerebral palsy, Rehabilitation, Motor control, Motor learning, Motor training, Physical therapy, Children

## Abstract

**Background:**

Cerebral palsy (CP) is the most common cause of physical disability in children. The best opportunity to maximize lifelong independence is early in motor development when there is the most potential for neuroplastic change, but how best to optimize motor ability during this narrow window remains unknown. We have systematically developed and pilot-tested a novel intervention that incorporates overlapping principles of neurorehabilitation and infant motor learning in a context that promotes upright mobility skill and postural control development. The treatment, called iMOVE therapy, was designed to allow young children with CP to self-initiate motor learning experiences similar to their typically developing peers. This manuscript describes the protocol for a subsequent clinical trial to test the efficacy of iMOVE therapy compared to conventional therapy on gross motor development and other secondary outcomes in young children with CP.

**Methods:**

The study is a single-blind randomized controlled trial. Forty-two participants with CP or suspected CP between the ages of 1–3 years will be randomized to receive either the iMOVE or conventional therapy group. Distinguishing characteristics of each group are detailed. Repeated measures of gross motor function will be collected throughout the 12–24 week intervention phase and at three follow-up points over one year post therapy. Secondary outcomes include measures of postural control, physical activity, participation and caregiver satisfaction.

**Discussion:**

This clinical trial will add to a small, but growing, body of literature on early interventions to optimize the development of motor control in young children with CP. The information learned will inform clinical practice of early treatment strategies and may contribute to improving the trajectory of motor development and reducing lifelong physical disability in individuals with CP.

**Trial registration:**

ClinicalTrials.gov identifier NCT02340026. Registered January 16, 2015.

**Electronic supplementary material:**

The online version of this article (10.1186/s12887-018-1303-8) contains supplementary material, which is available to authorized users.

## Background

An estimated 15 million people currently live with cerebral palsy (CP) worldwide. CP is the most common cause of physical disability in children [1] with a prevalence of over 3 cases per 1000 that has remained stable over recent decades despite advances in pre- and perinatal care [2, 3]. The degree of restriction in life participation in those with CP is predicted by the degree of physical disability, which varies widely from limitations in balance and coordination to full dependence on others for care [4]. This relationship between the severity of physical disability and participation restriction has been reported in infancy [5], childhood [6, 7], and adolescence and young adulthood [8]. Independent of cognitive impairment, the severity of physical disability in childhood is a predictor of independent living in young adulthood [9].

The best opportunity to maximize lifelong independence is early in motor development when the differences in motor skill between future functional levels are relatively small and there is the most potential for neuroplastic change. Gross motor ability typically plateaus by 4–7 years of age [10] in those with CP, after which motor ability is relatively fixed. In fact, the Gross Motor Function Classification System (GMFCS) [4] of motor severity remains relatively stable throughout childhood and adulthood [11, 12], regardless of treatment. However, the early years of life are an exception with less stability in GMFCS classifications before the age of 2 years [13]. There is growing evidence of a critical period of neuroplasticity for motor control centers in the brain. Recent work has confirmed that plasticity in the motor system is both activity-dependent, and more robust in early as compared to later years [14, 15]. Moreover, maladaptive plasticity is difficult to reverse once established [16]. These observations suggest that there is a window of opportunity for interventions applied *prior* to the developmental plateau to improve the trajectory of motor development in childhood and reduce lifelong physical disability.

Despite this evidence of an early critical period for neuroplasticity in motor control centers, there remains little application to individuals with CP and how best to optimize motor ability during this narrow window remains unknown. Treatments addressing secondary musculoskeletal impairments such as muscle and bone abnormalities in older children that develop in response to poor motor control remain among the most common treatment approaches for CP [17]. These interventions are important to manage the course of CP, but do not address the primary impairment of poor neural control of movement [18].

The most effective neuromotor rehabilitation programs in adults include *intensive*, *early*, and *challenging* motor practice [19–21], and these principles are supported by training-dependent plasticity in cortical structures [22–24]. Demonstrating *variability* in movement patterns reflects complex motor skill [25] and motor variability during rehabilitation also enhances motor outcomes [26, 27]. *Salience* is the meaningfulness of the training to the patient and promotes active engagement and facilitates neuroplasticity [28, 29]. Finally, the critical role of *error* in motor learning and rehabilitation has been increasingly recognized [30, 31], with diminished long-term gains when error is absent during practice [32].

It is perhaps no coincidence that many neurorehabilitation training principles are also important components of typical infant motor learning. Typical infant movement is characterized by a high degree of motor exploration [33], error [34, 35] and movement variability [36, 37], which are critical factors in the refinement of motor control. Young children with CP often cannot create these experiences on their own, losing natural opportunities to learn more coordinated movements and establish the associated neural pathways that control skilled movement. As a result, rehabilitation practice for these children does not always reflect the key learning principles of typical motor development, and is often more therapist-directed with minimal exploration, variability and error. In contrast to their typically developing peers, young children with CP repeatedly practice poorly-controlled motor patterns.

We have systematically developed and pilot-tested a novel intervention designed to allow infants and toddlers with CP to create for themselves motor learning experiences more similar to their typically developing peers [38]. We provide children with a minimal amount of support during the development of upright mobility skills, without constraining any movement. We use *dynamic* weight support technology as a tool to help create an environment that allows participants to practice motor skills that they are as yet unable, or otherwise may never learn, to do. This dynamic weight support system does not suspend the child in place and therefore does not constrain their movements, but continuously provides the desired amount of weight assistance, independent of where the child moves within the limits of an overhead track system. For example, the child can sit, stand, walk, ascend stairs, squat to reach the floor, turn around to move in the opposite direction, and even crawl, all while the system maintains constant weight support by controlling the variable length of the cable that joins the harness and track. The child’s movements are not restricted by the length of the cable (as in traditional static systems), and thus the system does not prevent trunk movement, but allows postural error, sway and falls while assisting all movements through weight support. The degree of weight support can be gradually reduced as the child’s coordination and motor control improve. With the dynamic weight support, they are able to practice challenging motor skills with less direction and physical support from a therapist. This approach, used in the context of guiding principles that promote exploration, variability and error during movement, allows toddlers with CP to have motor learning experiences through playful discovery similar to their typically developing peers.

The development and preliminary testing of the treatment, called iMOVE (Intensive Mobility training with Variability and Error), has been consistent with a stage model for behavioral therapies [39]. The treatment was designed to incorporate overlapping principles of neurorehabilitation and infant motor learning in a context that promotes upright mobility skill and postural control development. We conducted a single-subject research design pilot study to evaluate the safety, feasibility, and tolerability of the intervention, as well as the appropriateness of the primary outcome measure, in the target population. Five children (aged 12–27 months, GMFCS I-III) participated in the study with repeated measures of gross motor function during 6-week baseline and treatment phases, and after a 6-week follow-up phase. No adverse events occurred. Four of five children demonstrated gains in motor development during intervention that were 3.8 to 15.1 times their baseline rate. Additional details of the treatment development and feasibility testing have been described [38].

Prospective comparison to intensity-matched current rehabilitation intervention is needed to confirm the potential advantages of iMOVE treatment on motor development in young children with CP. We describe the protocol for the subsequent clinical trial in this manuscript. The trial is a single-blind randomized controlled trial comparing the outcomes of iMOVE therapy to dose-matched conventional physical therapy on gross motor development and other secondary outcomes in young children with CP. We hypothesize that participants who receive iMOVE therapy will make greater gains in motor development than participants who receive conventional rehabilitation, and that these gains will be maintained one year after treatment.

## Methods/Design

### Study design

The clinical trial is a single-blind, single-site randomized controlled, parallel groups trial to compare the outcomes of iMOVE therapy to dose- matched conventional physical therapy (CONV) on gross motor development in toddlers with CP. Secondary outcomes include measures of postural control, physical activity at home, engagement in daily life, and caregiver satisfaction. The intervention phase will be a minimum of 12 weeks, and participants can choose to extend the intervention to 18 or 24 weeks in duration. Repeated assessments of gross motor function and secondary outcomes will be administered during the 12–24 week intervention phase and at 3, 6, and 12-month follow-up points after treatment to track the developmental trajectory of motor function. An additional file includes the populated SPIRIT checklist of protocol components (see Additional file 1) [40].

### Aims

The primary aim is to compare changes from baseline in gross motor function between the iMOVE therapy and the CONV therapy. We hypothesize that participants who receive iMOVE therapy will make greater gains in motor development after 12 weeks (and after 18 and 24 weeks, as applicable) than participants who receive CONV rehabilitation, and that these gains will be maintained at each follow-up point (3, 6 and 12 months) after treatment. The secondary objective is to compare changes in postural control, physical activity at home, caregiver satisfaction and engagement in daily life between the iMOVE therapy and the CONV therapy. We hypothesize that participants who receive iMOVE therapy will make greater gains in postural control, physical activity at home, caregiver satisfaction and engagement in daily life after 12 weeks (and after 18 and 24 weeks, as applicable) than participants who receive CONV rehabilitation, and that these gains will be maintained at each follow-up point (3, 6 and 12 months) after treatment.

### Setting

The study will be conducted at a single site – The Children’s Hospital of Philadelphia, PA, USA, which is a large urban pediatric academic medical center. The majority of study visits will occur at the main campus, with occasional therapy sessions (estimated less than 10%) at one suburban satellite location as needed to increase convenience for participants.

### Study sample

Although most children who are later diagnosed with CP demonstrate clearly abnormal motor patterns or neurological signs in infancy, a definitive diagnosis of CP is sometimes not made until key motor milestones, such as independent walking, are significantly delayed. As a result, some children are not formally diagnosed until 18–24 months of age. Consistent with other work in the target population, we will define “suspected” CP as the combination of a motor delay with the presence of a neurological sign associated with CP, such as spasticity or periventricular leukomalacia (PVL) [41].

The selection criteria were developed with the goal to deliver the intervention during upright motor skill acquisition, and were refined by the outcomes of the pilot work. The wide heterogeneity in CP will be lessened in this sample by defining a window of pre-walking motor ability, defining a minimum level of cognitive function using a standard 12 month developmental milestone [42], and excluding children whose primary underlying neurological sign is hypotonia, which may be indicative of a neuromuscular disorder other than CP [43].

Eligible participants will meet the following criteria: 12–36 months of age, diagnosis of CP or suspected CP (motor percentile rank less than the 10th percentile on the Bayley Scales of Infant Development [44, 45], and a neurological sign associated with CP, such as spasticity), the ability to initiate pulling to stand at a surface as indicated by a score of 1 on the Gross Motor Function Measure (GMFM) item 52 [46], and the cognitive ability to follow one-step commands. Participants will be ineligible for the trial if they demonstrate any of the following: secondary orthopedic, neuromuscular or cardiovascular condition unrelated to CP, general muscle hypotonia without other neurological signs associated with CP, independent walking ability as indicated by a score of 3 on GMFM item 69, or history of surgery or injury to the lower extremities in the past 6 months.

### Sample size estimation

Predicted change in the iMOVE group was estimated from data collected from four children (pilot study data) who would meet the proposed inclusion criteria. A mean GMFM-66 [47] increase of 5.3 was observed after 6 weeks of treatment. We estimated that a change of 10.6 would be expected within 24 weeks of treatment. Predicted change in the CONV group was determined from published GMFM-66 percentile scores for average change over six months’ time [48]. We are planning to recruit a total of 42 participants (21 per treatment group). We estimated a uniformed attrition rate of 20% by the end of the study, therefore, evaluable data from 34 participants (17 per group) will be available. With a sample size of 34, a 2-sided 95% confidence interval for the estimated difference in GMFM-66 between the two interventions will extend +/− 6 units from the observed difference assuming a conservative standard deviation of 9.

### Recruitment

The primary avenues for recruitment will be through the Neonatal Follow-up and Cerebral Palsy programs at CHOP. Eligible patients receiving outpatient therapy services at the Center for Rehabilitation will also be invited to participate. Additional candidates who are not CHOP patients will be recruited through mailings to local physical therapists and occupational therapists. All recruitment materials will receive prior ethics approval.

### Screening and randomization

Candidate screening will be conducted by the primary research therapist using the inclusion and exclusion criteria. Screening will include medical record review, physical therapy examination, and administration of the motor subscale of the Bayley Scales of Infant Development (BSID-III) [45]. The parent or legal guardian will provide written informed consent prior to the start of any study activities. Written assents of minors will not be obtained due to the age of the participants.

After the initial Gross Motor Function Measure (GMFM-66) score from Assessment 1 is obtained, participants will be randomized to either the iMOVE or CONV treatment group, using a randomization scheme designed by the study statistician to stratify participants by baseline motor ability and age. A study team member not involved in the screening of candidates or the delivery of interventions will assess a secure electronic file to determine group assignment prior to the first therapy visit. The randomization scheme will ensure equivalence between groups in motor ability and age at baseline. Allocation ratio to either of the two groups is 1:1. Blinding of participants to treatment group is not feasible with the proposed interventions. A table of study procedures is depicted in Table 1.

**Table 1:**
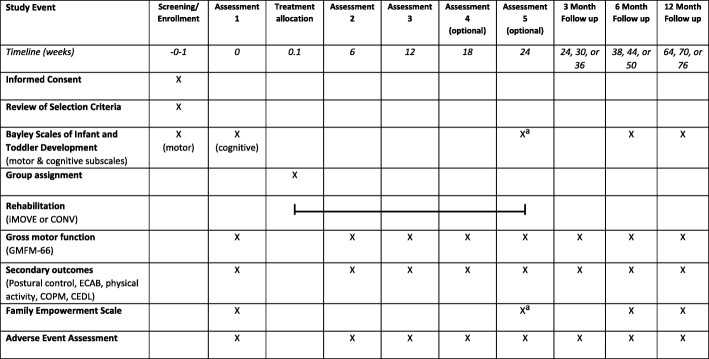
Schedule of Study Procedures

*GMFM-66* Gross Motor Function Measure, *ECAB* Early Clinical Assessment of Balance, *COPM* Canadian Occupational Performance Measure, *CEDL* Child Engagement of Daily Life

^a^to be conducted at the post-treatment assessment, which may be Assessment 3 (12 week) or 4 (18 week)

### Interventions

Treatment will start within one week after the baseline assessment. All treatment sessions will be delivered by experienced pediatric physical therapists. Training materials will be prepared for therapist training and to serve as a resource for the distinguishing characteristics of each group to assure consistency in delivery of therapy within each group. Study therapists will participate in a half-day training workshop, supplemented by video review of pilot study sessions. Therapists will maintain a training log for each session, describing general activities, and the amount of weight support for participants in the iMOVE group. One session per week will be videotaped (if separate parental consent is provided) for later coding of therapy activities to relate the content of therapy sessions to outcomes. Randomly selected videos will be used for periodic checks to ensure treatment fidelity, specifically that activities in the groups remain different, and are consistent with the distinguishing characteristics of each group. To encourage adherence, caregivers of participants will be modestly compensated for each assessment session completed, and some travel costs will be covered for each visit.

Therapy in each group will be delivered 3 times per week for 30 min each session. The intensity of treatment (90 min per week) in the proposed study will approximate the average amount of physical therapy received by young children with CP in the United States. The average amount of physical therapy is 82 (SD 60) minutes per week in the United States, and 90 (SD 60) minutes per week in the Philadelphia metropolitan area [49]. The 90 min per week of either treatment in the proposed study reflects this current intervention practice. However, the wide variability in the standard amount of services means that not all children would receive this intensity outside of the research study. This variability in current practice is a common issue in identifying “standard of care” in rehabilitation trials. As such, it has been determined in gold-standard trials that matching the treatment intensity of the experimental group is the most important component of the “control” group [21, 50], and our approach reflects this standard.

Children will be able to continue their outside therapies, if their families’ choose to do so. Whether they reduce or continue their pre-enrollment therapy schedule, families will be asked to maintain the schedule of outside physical therapy constant throughout the treatment phase. It is anticipated that most children will be receiving at least early intervention therapy services in the home. Other medical care will likewise not be restricted but will be recorded.

#### iMOVE therapy

The experimental therapy group will receive dynamic weight support (using the ZeroG® Gait and Balance training system, Aretech LLC, Ashburn, VA) during all therapy time, and the environment will be arranged to encourage active motor exploration by the child, in order to promote the motor variability, exploration, and error experiences that characterize the typical development of upright motor skills and walking. Activities will be graded in difficulty to the child’s ability and will include: moving between the floor and standing, walking, squatting to reach the floor, climbing/walking up and down steps and inclines, and other typical toddler movements. The therapist will minimally assist the child as needed to perform the movements he/she initiates. See Table 2 for the distinguishing characteristics of the iMOVE therapy group.

**Table 2:** Distinguishing characteristics of the iMOVE and CONV therapy groups

iMOVE therapy	CONV therapy
● Dynamic weight support ● Child-directed (child initiates activities) ● No assistive devices, limited use of orthoses, no treadmill (toddler-salient environment only) ● Encourage high degree of error with reduced physical assistance ● Encourage frequent variability in motor tasks (no redirection when moving from one activity to another) ● Physical therapist expertise is focused on designing a salient and challenging environment for the child’s specific interests and ability level to encourage engagement, variability, challenge, and error experience, and on determining the appropriate amount of weight assistance	● No or static weight support ● Therapist-directed (therapist initiates) ● Traditional early gait training methods: use of assistive devices/orthoses and may use treadmill ● Focus on producing “typical” movement patterns with extensive manual guidance/correction from therapist, prevention of falls ● Therapy activities grouped into blocks of practice (i.e. repeated floor to stand practice followed by gait training) ● Physical therapist expertise is focused on designing and directing the specific practice activities each session, tailored to the individual child

The floor area within 3 ft below either side of the overhead track for a distance of approximately 20 ft (approximately 120 ft^2^ total) will be defined with colorful thin rubber interlocking mats and arranged with pediatric toys and activities, tailored to the child’s interests and to encourage motor skills just beyond his/her current ability level. The dynamic support system continuously provides a constant amount of weight assistance (as determined by the therapist) by controlling the length of the cable joining the harness and track and by moving along the overhead track as the user moves about the space (i.e. cable lengthens if child moves to the floor and shortens if child climbs up steps, with no lag time). The child’s movements will not be restricted at all within this space. This arrangement works well to keep children within the limits of the overhead track and provide ample opportunity and space for motor play and exploration.

The initial amount of weight assistance will be determined by the level that allows walking and squatting to reach the floor with the least amount of assistance from the therapist, up to a maximum of 50% of the child’s weight. Weight assistance will be gradually reduced during the treatment phase as postural control and coordination improve.

#### Conventional therapy (CONV)

The conventional therapy group will receive traditional, therapist-directed pediatric physical therapy at the same frequency as the iMOVE group. Therapy will focus on early gait training strategies and encouragement of “normal” movement patterns for walking and other age-appropriate movements, with manual guidance or correction of atypical movements from the therapist. This group may use assistive devices, orthoses, and may occasionally receive static body weight support for gait training. Examples include: using a posterior rolling walker with ankle foot orthoses (braces), physically guided practice of standing from the floor through half kneeling, manual correction of side steps while cruising at a bench, and repeated sit to stand practice from a small chair. Therapy activities will be performed in blocks of practice, with the specific activities and level of therapist assistance tailored to each child. See Table 2 for the distinguishing characteristics of the CONV therapy group.

### Outcome measurement

A blinded assessor who is an experienced pediatric physical therapist will collect all outcomes measures. Any unintentional unblinding will be recorded and reported with the results. Assessments will be conducted every six weeks through 24 weeks after therapy begins, and at three follow-up points (3, 6, and 12 months) after the end of treatment. The *primary outcome* is the GMFM-66, a Rasch-analyzed measure of gross motor function designed for children with CP [47]. Computation of the total score involves statistical weighting of the raw item scores for difficulty, with calculation of a standard error of measurement (SEM). This SEM is essentially a measure of the confidence in the accuracy of the score, with low values reflecting greater confidence in the score. The average SEM for all GMFM-66 scores in the pilot study was 1.16 (range of 1.05–1.47) reflecting excellent confidence in the accuracy of the scores for participants from the target population. The blinded assessor will be trained for reliability with videos from the pilot study.

#### Secondary outcomes

Include measures of postural control, physical activity at home, caregiver satisfaction and participation. Postural control will be measured by the Early Clinical Assessment of Balance [51, 52], which was designed to measure postural control in young children with physical disabilities, and by the sample entropy of seated center of pressure data [25, 53]. Center of pressure data will be collected using a computerized posturography system with embedded force plate (Neurocom SMART Balance Master, Natus Medical Inc.), and with video synchronization for verification of data integrity. Participants will maintain static sitting on the force platform without reaching with the upper extremities or rocking with the trunk for several 10–20 s trials. Time series data will be processed with signal processing software first using surrogation methods to verify that nonlinear methods are appropriate, and then to determine the sample entropy. The sample entropy is a measure of regularity, or predictability, in a time series that when applied to center of pressure data, indicates the level of complexity of postural control [53]. Physical activity at home will be measured by a wearable inertial sensor (Sapphire sensor, APDM, Inc., Portland, OR) worn on the dominant thigh during floor play time at home. A tri-axial accelerometer in the sensor will record data at 128 Hz. Caregivers will record several bouts of floor play time over one-week and indicated the date, start and stop times on a log. Time-normalized user acceleration will be calculated using signal processing software and will serve as a proxy measure of self-initiated physical activity. Caregiver satisfaction will be measured with the Canadian Occupational Performance Measure [54]. The same caregiver of each participant will rate their child’s performance and satisfaction on the caregiver’s pre-identified goals at each assessment session. Participation will be measured by the Child Engagement in Daily Life [55], a caregiver-proxy measure of participation designed for young children with disabilities. The same caregiver of each participant will complete questions about the child’s frequency of and enjoyment with various activities at each assessment session.

#### Treatment modifiers

Measures of cognition and caregiver self-efficacy will be collected periodically as known modifiers of response to rehabilitation, which may contribute to variability in outcomes [56, 57]. Cognition will be measured by the BSID-III cognitive subscale [45]. To avoid a learning effect from repeated testing, this will be completed only every six months. Caregiver self-efficacy will be measured by the Family Empowerment Scale [58]. The same caregiver of each participant will complete the questionnaire every six months.

### Subject completion/withdrawal

Subjects may withdraw from the study at any time without prejudice to their care. Intent to treat procedures will be followed such that participants will not be withdrawn from the study by the investigators for missing treatment sessions. Participants who withdraw from the study will have all procedures enumerated for Assessment 5 completed as the early termination visit, if possible.

### Adverse event reporting

The study procedures present no more than minimal risk to participants, and as such serious adverse events are not expected. If any unanticipated problems related to the research involving risks to subjects or others happen during the course of this study, they will be reported to the IRB. Adverse events that are not serious but that are notable and could involve risks to participants will be summarized in narrative or other format and submitted to the IRB at the time of continuing review.

### Data management

All data and records generated during this study will be kept confidential in accordance with institutional policies and HIPAA on subject privacy and the Investigator and other site personnel will not use such data and records for any purpose other than conducting the study. Participants will be assigned a unique identifier that contains no protected health information. Access to all data will be controlled by the PI. No identifiable data will be used for future study without first obtaining IRB approval. We will archive our video and related metadata, as permitted by individual participants, in Databrary, the NIH- and NSF-funded web-based video repository for developmental behavioral science to share video for reuse and education among developmental scientists [59]. The investigator will obtain a data use agreement between the provider (the PI) of the data and any recipient researchers (including others at CHOP) before sharing other study datasets.

Hard copies of case report forms and source data will be stored in a locked cabinet in a locked office. Electronic source data will be stored on a network share drive with access controlled by the principal investigator. All data will be entered and stored in a project-specific REDCap (Research Electronic Data Capture) database [60]. The database will be password-protected and daily backups will be stored. It will incorporate range checks and between-variables consistency checks to ensure quality control. There will be double data entry of the primary and secondary outcomes by a specially trained individual external to the study operations team.

### Data monitoring

The incidence of adverse events is expected to be low in this single-site minimal risk research. The principal investigator will be responsible for monitoring the data and safety of all participants. In addition to obtaining ethics approval and the data management procedures outlined above, the principal investigator will hold biweekly study team meetings to evaluate the safety and progress of all research procedures. Standard procedures for all data collection methods will be reviewed at the start and periodically throughout the study. Data checks for errors will be performed prior to analysis. Videos will be reviewed regularly to ensure that the rehabilitation programs are delivered as intended. Unexpected safety concerns will be communicated with the IRB and funding sponsor, and if adverse events occur in more than 15% of participants, we will appoint a Study Monitoring Committee to review and monitor safety for the remaining duration of the study.

### Statistical analysis

The full analysis set (FAS) includes all randomized patients. Efficacy of treatment analyses will be based on the treatment allocated at randomization (as randomized). The per protocol set (PPS) includes all patients in the FAS except for those who are excluded by protocol violations that affect the interpretation of study results. The primary endpoint, gross motor function, will be evaluated on the FAS and PPS. Treatment compliance/administration and safety events will be analyzed using the FAS. Baseline characteristics for the total sample and by treatment group and by treatment periods will be summarized by standard descriptive summaries (including mean, standard deviation, median, minimum, maximum and range for continuous variables and frequency counts and percentages categorical variables). We will also report the 95% confidence interval for pertinent means and proportions. Baseline characteristics in each group will be compared using two-sample tests, including t-tests or the Mann-Whitney (non-parametric) tests for continuous variables, and the chi-square tests for categorical variables. For the analysis of the primary outcome, we will use a univariate approach including analyses of variance and covariance to compare changes from baseline to post in GMFM-66 scores between participants receiving iMOVE therapy and those receiving CONV therapy. The primary efficacy analysis will occur after 12 weeks of intervention. Outcomes after 6, 18, and 24 weeks, and during the follow-up year, will be compared in a similar fashion to understand the dose-response trajectories of each intervention. We will also use a multivariate approach using linear mixed effects model [61] or the Generalized Estimating Equation (GEE) [62]. The advantage of using the mixed effects model or the GEE approach is that they will not drop subjects from the analysis due to not having measurement at any of the post-treatment time points. Also, such analyses will allow us to examine the between subjects effects which represent a factor with two levels (treatment conditions) and within subjects effects which represent time effects (pre and post measurements) and a time by condition interaction. Cognition and caregiver self-efficacy will be included as covariates in these analyses. Similar procedures will be used for the analysis of secondary outcomes, with appropriate tests for parametric (sample entropy of center of pressure, physical activity) and non-parametric measures (Early Clinical Assessment of Balance, caregiver satisfaction, Child Engagement in Daily Life). We will report the *p* values associated with each of the statistical tests.

## Discussion

This clinical trial will add to a small, but growing, body of literature on early interventions for infants and toddlers with CP or suspected CP [63, 64]. While the study design of a flexible treatment duration (12, 18, or 24 weeks) introduces statistical complexity, it will allow a standard analysis at the primary 12-week endpoint as well as valuable dose-response information, which will inform the design of future work. This design also mimics clinical practice with episodes of rehabilitation services delivered until participants achieve a goal or a plateau, rather than assigning an arbitrary treatment duration in advance. The information learned will be valuable in increasing our understanding of how best to optimize the potential of the developing brain to support motor function after injury. This understanding will inform clinical practice and may contribute to improving the trajectory of motor development and reducing lifelong physical disability in individuals with CP.

## Additional file

Additional file 1: SPIRIT 2013 Checklist. (DOC 122 kb)

## Abbreviations

BSID-III: Bayley Scales of Infant Development, Third Edition
CONV: Conventional therapy
CP: Cerebral palsy
GEE: Generalized Estimating Equation
GMFM: Gross Motor Function Measure
iMOVE: Intensive Mobility training with Variability and Error therapy
IRB: Institutional Review Board
PVL: Periventricular leukomalacia
SEM: Standard error of measurement
